# Differential Diagnosis of Prostate Cancer Grade to Augment Clinical Diagnosis Based on Classifier Models with Tuned Hyperparameters

**DOI:** 10.3390/cancers16112163

**Published:** 2024-06-06

**Authors:** Saleh T. Alanezi, Marcin Jan Kraśny, Christoph Kleefeld, Niall Colgan

**Affiliations:** 1 Department of Physics, College of Science, Northern Border University, Arar P.O. Box 1321, Saudi Arabia; 2 Department of Physics, School of Natural Sciences, College of Science and Engineering, University of Galway, H91 TK33 Galway, Ireland; m.j.krasny@nuigalway.ie (M.J.K.); christoph.kleefeld@universityofgalway.ie (C.K.); niall.colgan@tus.ie (N.C.); 3 Translational Medical Device Lab (TMDLab), Lambe Institute for Translational Research, University of Galway, H91 V4AY Galway, Ireland; 4 Faculty of Engineering & Informatics, Technological University of the Shannon, N37 HD68 Athlone, Ireland

**Keywords:** prostate cancer, multiparametric (mp-MRI), machine learning

## Abstract

**Simple Summary:**

Multiparametric MRI with radiomics features derived from T_2_WI and ADC maps distinguished non-tumor regions from significant cancer and predicted the Gleason score using support vector machine (SVM) and random forest (RF) classification methods with tuned hyperparameters, as well as recursive feature elimination (RFE) and the least absolute shrinkage and selection operator (LASSO) feature selection methods. Successful application of a novel approach to machine learning incorporating recursive feature elimination combined with random forest and support vector classifiers allowed stratification of Gleeson scores in clinical cohorts at a sensitivity greater than 0.91.

**Abstract:**

We developed a novel machine-learning algorithm to augment the clinical diagnosis of prostate cancer utilizing first and second-order texture analysis metrics in a novel application of machine-learning radiomics analysis. We successfully discriminated between significant prostate cancers versus non-tumor regions and provided accurate prediction between Gleason score cohorts with statistical sensitivity of 0.82, 0.81 and 0.91 in three separate pathology classifications. Tumor heterogeneity and prediction of the Gleason score were quantified using two feature selection approaches and two separate classifiers with tuned hyperparameters. There was a total of 71 patients analyzed in this study. Multiparametric MRI, incorporating T_2_WI and ADC maps, were used to derive radiomics features. Recursive feature elimination (RFE), the least absolute shrinkage and selection operator (LASSO), and two classification approaches, incorporating a support vector machine (SVM) (with randomized search) and random forest (RF) (with grid search), were utilized to differentiate between non-tumor regions and significant cancer while also predicting the Gleason score. In T_2_WI images, the RFE feature selection approach combined with RF and SVM classifiers outperformed LASSO with SVM and RF classifiers. The best performance was achieved by combining LASSO and SVM into a model that used both T_2_WI and ADC images. This model had an area under the curve (AUC) of 0.91. Radiomic features computed from ADC and T_2_WI images were used to predict three groups of Gleason score using two kinds of feature selection methods (RFE and LASSO), RF and SVM classifier models with tuned hyperparameters. Using combined sequences (T_2_WI and ADC map images) and combined radiomics (1st and GLCM features), LASSO, with a feature selection method with RF, was able to predict G3 with the highest sensitivity at a level AUC of 0.92. To predict G3 for single sequence (T_2_WI images) using GLCM features, LASSO with SVM achieved the highest sensitivity with an AUC of 0.92.

## 1. Introduction

Prostate cancer is the second predominant male tumor globally, with 1,276,106 new cases and 358,989 deaths in 2018 [1,2]. That is 7.1% of new cases and 3.8% of all male cancer mortality in 2018 [3]. Globally, the median age for detection of prostate cancer is 66 years old, and both the recurrence and fatality rates rise with age [4,5]. Early detection of tumors increases the chances of being cured because treatment works even if the cancer is localized.

Multiparametric magnetic resonance imaging (mp-MRI) has been used extensively in prostate cancer (PCa) scanning, identification, and grading throughout the last few decades [6,7]. It is possible to obtain high-resolution anatomical and functional images using the mp-MRI imaging technique [8]. T_1_ weighted images (T_1_WI) and T_2_ weighted images (T_2_WI) are anatomic sequences used in multiparametric prostate MRI. For example, the zonal structure and tumor foci cannot be identified using T_1_WI. It is possible to employ T_1_WI to discover biopsy-associated haemorrhage, which can interfere with the capacity of other PCa MRI techniques to provide accurate diagnoses. T_2_WI provides the best soft-tissue imaging for malignancies, zonal morphology, seminal vesicle (SV), anterior fibromuscular stroma (AFS), neurovascular bundles, and the capsule [9]. Diffusion-weighted imaging (DWI), Magnetic resonance spectroscopic imaging (MRSI), and Dynamic contrast-enhanced (DCE) are functional MRI sequences [10]. The DWI technique was developed and implemented to detect an acute cerebrovascular stroke. DWI compares water diffusion in soft tissues and free solution to produce image contrast. When a PCa grows, there is a growth in cellularity and degradation of ductal architecture, which limits fluid flow through the prostate [11]. The b-value and Apparent Diffusion Coefficient (ADC) are the two types of images used for analysis in DWI. Tumor diagnostic outcome is improved by utilizing b-values between 1400 and 2000 [12,13,14]. Clinical interpretation from DWI is subjective; nevertheless, water molecules’ limitations may be measured quantitatively. Interpretation is performed with ADC maps and ADC measurements (mm^2^/s). and ADC levels and Gleason scores are proportionally related [15,16]. By using a machine learning approach with clinically relevant radiomics metrics as inputs we aim to improve the interpretation and augment clinical diagnosis.

Radiomics can generate (200+) statistical variables from medical images automatically. Patient anatomy can significantly vary in shape and texture depending on the imaging technique used [17]. Using automated or semi-automated radiomic metrics we could improve diagnostic accuracy. Textural analysis has been used to extract tissue information from medical images since the 1980s [18,19]. It recognizes that intratumor heterogeneity has significant implications for cancer research, which could be represented by tumors’ texture [20,21]. Radiomics relies heavily on texture analysis (TA), a necessary part of the process [21,22]. Radiomics is the technique used to collect essential and extensive data from clinical images and give variables that can be used to assist in detection, prognostic, and treatment response [22,23,24,25,26,27]. When developing a radiomics model, selecting the best Machine Learning (ML) model is key and different ML approaches may perform differently when applied to different tissues [27,28,29,30,31,32,33].

Training is used to derive many of the algorithm parameters used by machine learning (ML), and most contemporary ML algorithms must tune parameters to improve feature identification referred to as Hyperparameters [34,35,36]. The hyperparameters are fine-tuned to optimize an algorithm for a specific learning task [37]. Hyperparameter optimization usually employs Grid and Random Search techniques [38]. Grid Search is a method using all possible permutations of hyperparameters. The training data and the number of layers can be adjusted in a grid search as hyperparameters [39]. In contrast to a grid search, randomized search does not perform a comprehensive investigation of the hyperparameter space. Nonetheless, it permits us to investigate a wider variety of hyperparameter value settings more effectively and affordably [39]. Weerts et al. [37] stated that an increased tuning risk and relative tuning risk were observed from the random forest’s max features and SVM’s gamma and C, suggesting that it is essential to tune these hyperparameters. In the domain of prostate cancer classification and grading, many prior studies have applied machine learning techniques with default hyperparameters, often without extensive hyperparameter optimization. In contrast, our research distinguishes itself by prioritizing hyperparameter tuning. This deliberate optimization process enhances the precision and reliability of our ML models, contributing to great precision in clinically relevant results. Our work aims to advance the field by systematically refining the parameters that underpin the diagnosis of prostate cancer.

The study aimed to use different classifiers (with tuned hyperparameters) and two feature selection methods (i.e., to find the best for classification and prediction). Multiparametric MRI-derived radiomics features were used (including T_2_WI and ADC map images). First, to quantify tumor heterogeneity between significant versus non-tumor regions. Second, to predict Gleason scores (i.e., G2 = 3 + 4; G3 = 4 + 3; and G4 = 4 + 4 = 8, 3 + 5 = 8 (G4), 9 (G5) or 10 (G5)) for significant prostate cancer.

## 2. Materials and Methods

### 2.1. Patient Group

This study utilized a dataset from The Cancer Imaging Archive (TCIA) funded through the SPIE, NCI/NIH, AAPM, and Radboud University [40]. The population set used in this work consists of 99 patients, including T_2_WI and Apparent Diffusion Coefficient map (ADC) series from the open-source, freely released SPIE-AAPM-NCI PROSTATEx-2 [34]. the total number of patients (*n* = 99); received a 3T mp-MRI using a body coil. *n* = 29 The patients had a non-significant tumor at (3 + 3) Gleason grade excluded; (e) *n* = 71 patients had a significant tumor (≥3 + 4) at Gleason grade (GS). 

Images were obtained using a Siemens 3T MRI techniques (MAGNETOM Skyra, Siemens Healthcare, Erlangen, Germany) utilising a pelvic phased-array coil. The axial T_2_WI and ADC maps were employed for imaging assessment. The current clinical practice uses a T_2_WI and a minimum of one if not two functional approaches (e.g., DCE, and spectroscopic) are used to identify prostate cancer [8,41,42]. For precise localization, all biopsies were done under MR monitoring. A pathologist then rated biopsy specimens, which served as the ground truth. T_2_WI was obtained using a turbo spin echo sequence with 0.5 mm resolution and 3.6 mm slice thickness. The Diffusion weighted images were obtained using a single-shot echo-planar imaging procedure utilizing 2 mm in-plane resolution, 3.6 mm slice thickness, and three-dimensional diffusion encoding gradients. The scanner program generated the ADC map from three b-values (50, 400, and 800 s/mm^2^). Table 1 contains a description of the mp-MRI acquisition settings. The images were collected with no endorectal coil in line with PI-RADS recommendations for prostate MRI images [41].

### 2.2. Segmentation

**Figure 1:**
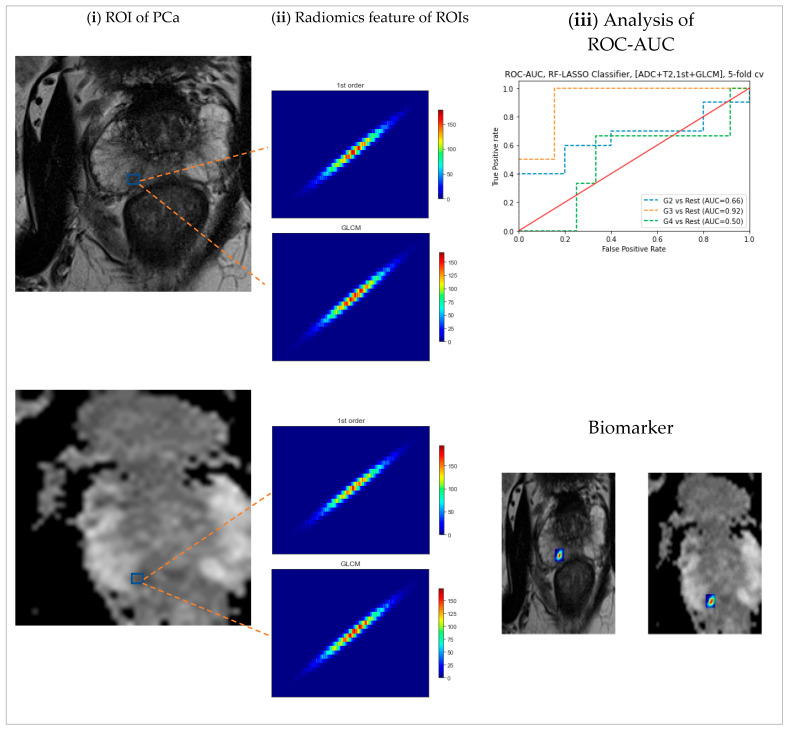
The prostate cancer classification approach entails three primary steps: (**i**) utilizing Regions of Interest (ROIs) that correspond to cancer locations on histology slides and MRI, specifically T2 weighted images and Apparent Diffusion Coefficient map images from 71 subjects; (**ii**) extracting both 1st and 2nd orders features; and (**iii**) conducting ROC-AUC analysis, which includes the generation of ROC curves.

Regions of interest (ROIs) for significant cancer were segmented manually from T_2_WI, and ADC images predefined ROIs from PROSTATEx-2 Challenge that is available on TCIA [40,43]. The LIFEx package was used for the segmentation process [20]. Non-tumor regions cancers segmented depending on the same region for significant cancer (in different regions) assessed for every subject’s lesion. Figure 1 illustrates a typical malignancy cancer segmentation on mp-MRI.

### 2.3. Feature Extraction

**Figure 2:**
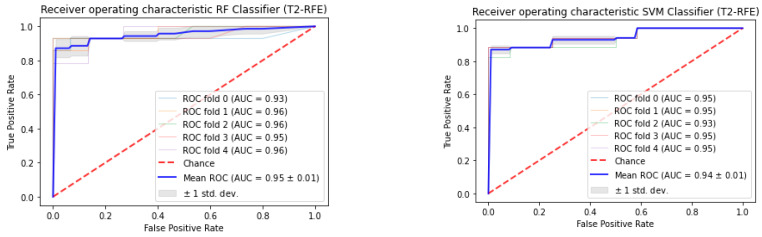
The classification of prostate cancer as significant versus non-tumor regions depends on RFE using mp-MRI within a 5-fold cross-validation.

Pre-processing, including intensity normalization and spatial resampling, was conducted for all mp-MRI images using LIFEx to derive radiomics features. The dimensions were rescaled to 0.5 × 0.5 × 3 mm, preserving the dataset’s in-plane and inter-plane resolutions. The radiomics features uniformity achieved using grey-level discretization defined between 1 and 128 bits/pixel. Absolute resampling between the minimum and maximum fixed bounds for all ROIs used for intensity resizing parameters [20]. Figure 2 demonstrates the analysis procedures. For each ROI, (a) 5 features were computed from the histogram, and (b) Six features were computed from grey-level features co-occurrence, leading to 11 features per ROI for each patient.

### 2.4. Feature Selection

Feature selection refers to the process of selecting essential features in predictive models. Irrelevant features can degrade the prediction model by contributing little to it [6]. Model overfitting challenges arise when there are too numerous features in the algorithm. A significant feature containing fewer numbers, but high precision can be minimised by determining the size of the feature set through the feature selection approach [44]. It is popular to use recursive feature elimination (RFE) [31,45,46,47,48] and to select the best features from the dataset. The least absolute shrinkage and selection operator (LASSO) and RFE were employed in this study for feature selection due to their high performance and widespread use. The Python environment with scikit-learn (version 1.0.2) was used to implement these feature selection algorithms.

#### Classification and Prediction

Both support vector machine (SVM) with hyperparameter tuning via grid search [45,48,49,50] and random forest (RF) with hyperparameter tuning via a randomized search [30,51] were used to achieve optimal and fit classification performance for significant cancer versus non-tumor regions and tuning hyper using the scikit-learn library from Python (1.0.2). These classification techniques were selected and assessed because they have been extensively used to identify different organs, as mentioned in previous studies [28,45,47,52]. To identify regions of significant cancer we employed radiomics parameters based on statistical features from both the 1st and 2nd order, derived from the Gray-Level Co-occurrence Matrix (GLCM). Our approach involved utilizing two ML classifiers: the Support Vector Machine algorithm and RF algorithm. For the RF model, we conducted a randomized search to fine-tune its hyperparameters, which encompassed factors such as the number of estimators, criterion, max depth, and max features. In contrast, for the SVM model, we engaged in a grid search method to optimize hyperparameters such as C, gamma, and the choice of kernel function.

To assess the effectiveness and dependability of these models, we carried out a K-fold cross-validation (CV) procedure with K adjusted to 5. The meticulous validation process ensured that the models we developed were able to accurately differentiate between areas with significant cancer and non-tumor regions based on radiomics statistics.

In order to predict outcomes within GS cohorts, radiomics parameters, specifically those relating to the first and second orders of the GLCM, were utilised as feed for a RF classifier with randomized search as well as an SVM classifier with hyperparameter tuning through grid search. The intention was to demonstrate the statistical significance of these parameters. We trained the Random Forest model using a randomized search method with various hyperparameter settings, including number estimators, criterion, max depth, and max features.

The SVM model with grid search trained with different hyperparameter settings (including c, gamma, and kernel). Then the models were computed using starfield K-fold cross-validation (k = 5). The RF and SVM-based tuning hyperparameter classifiers were interpreted using a binary classification method, with G2 vs. rest, G3 versus rest, and G4 versus rest employed to illustrate the AUC-ROC. Because of class imbalance, a classifier’s performance may suffer if all of the datasets are assigned to the majority class, leading to high accuracy in classification but low specificity or sensitivity [53]. Several ways to deal with this issue are through oversampling [54] and sample weighting [55]. To clarify the operation of one vs rest worked, we classified G2 from G3 and G4 using ROC-AUC as a binary classification. The G3 and G4 areas under curves were calculated utilizing the same approach (one vs. rest). This study used Python’s scikit-learn (v. 1.0.2) library to verify model validity using a five-fold cross-validation approach.

### 2.5. Statistical Analysis

Each radiomics parameter was tested for significance using the Kruskal-Wallis technique. Radiomic features and PCa patients’ significant cancer versus non-tumor regions were correlated using Spearman correlation. Statistical significance was determined using the Holm-Bonferroni method at a *p*-value of ˂0.05 [56].

Using the Kruskal-Wallis test, each radiomics feature was looked at again to see if it was significant in the GS cohorts. The value of the correlation between radiomics characteristics and the GS groups for prostate cancer subjects was determined using the Spearman correlation, which was employed to measure the correlation value. Statistical significance was determined using the Holm-Bonferroni method at a *p*-value of ˂0.05 [56].

## 3. Result

### 3.1. Patients

This study used prostate cancer subjects from the SPIE, NCI/NIH, and AAPM PROSTATEx-2 Cancer Imaging Archive (TCIA). To reflect the GS, subjects were categorized as follows: 39 subjects, G2 = 3 + 4; 18 subjects, G3 = 4 + 3; and G4 = 4 + 4 = 8; 13 subjects, 3 + 5 = 8, 9 (G5), or 10 (G5) more accurately. 

### 3.2. Relation between Radiomic Attributes and Significant versus Non-Tumor Regions

Each prostate cancer patient’s T_2_WI and ADC map images were used to extract radiomics features. The Kruskal-Wallis approach was used to ascertain if any feature from radiomics had statistical significance to make a comparison between significant tumor versus non-tumor regions. The radiomics features correlated with significant cancer versus nontumor regions using Spearman correlation.

In the Kruskal-Wallis significance test, eleven features were statistically significant: skewness, kurtosis, entropylog10, entropylog2, uniformity, jointentropylog10, jointentropylog2, correlation, contrast, dissimilarity, and angular second moment after applying the Holm-Bonferroni correction (Table 2). The Spearman correlation among radiomics attributes in significant cancer versus non-tumor regions reveals strong and significant correlation values of 0.31, 0.30, −0.33, −0.23, 0.37, 0.46, 0.56, 0.56, −0.25, 0.27, 0.26, 0.76, 0.80, and 0.37 for skewness, kurtosis, entropylog10, entropylog2, uniformity, jointentropylog10, jointentropylog2, angular second moment, contrast, dissimilarity, and correlation, respectively (Table 3).

### 3.3. Classifiers and Feature Selection Performance

The radiomics features were fed into a model that used RF and SVM classifiers with tuned hyperparameters to distinguish between significant cancer versus non-tumor regions in 71 PCa patients. For T_2_WI images, the RFE combined RF Classification algorithm obtained the maximum AUC of 0.95 ± 0.01 (with 5-fold CV). Furthermore, the RFE combined the SVM classification algorithm obtained the second maximum AUC of 0.94 ± 0.01 (with 5-fold cross-validation). Nevertheless, the feature selection technique LASSO using support vector machine classifier obtained the maximum AUC of 0.93 ± 0.01 (with 5-fold cross-validation). Furthermore, the selection technique LASSO combined random forest obtained the second maximum AUC of 0.88 ± 0.02 (with 5-fold CV). The LASSO combined SVM Classification algorithm obtained the maximum AUC of 0.89 ± 0.00 (with 5-fold CV) for ADC images. 

**Figure 3:**
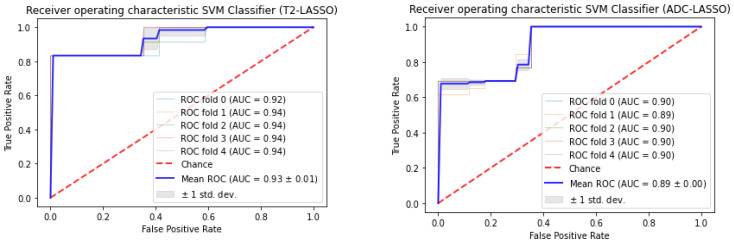
The classification of prostate cancer as significant versus non-tumor regions depends on LASSO using mp-MRI within a 5-fold cross-validation.

The feature selection approach LASSO combined RF classification algorithm also obtained the second maximum AUC of 0.89 ± 0.02 (with 5-fold CV). Nevertheless, the selection approach recursive feature elimination combined random forest classification algorithm obtained the maximum AUC of 0.85 ± 0.02 (with 5-fold cross-validation). Furthermore, the RFE combined SVM selection technique obtained the second maximum AUC of 0.84 ± 0.01 (with 5-fold CV). We selected the most appropriate feature selection technique and classification algorithm, Figure 2 and Figure 3 depict the significant receiver operating characteristic area under curves (ROC-AUC) for T_2_WI and ADC map images, respectively.

For combined sequences (T_2_WI and ADC map images), the LASSO combined SVM Classification algorithm obtained the maximum AUC of 0.91. RFE combined with the RF classification algorithm obtained the second maximum AUC of 0.88. For combined sequences (T_2_WI and ADC map images), the RFE combined SVM Classification algorithm obtained an AUC of 0.81and LASSO combined RF classification algorithm obtained an AUC of 0.84. 

### 3.4. Relationship between GS and Radiomics Attributes

The Kruskal-Wallis approach was used to ascertain if any feature from the radiomics aspect had statistical significance to make comparisons between the GS groups after retrieving radiomics features from T_2_WI and ADC map images of every prostate cancer subject. The radiomics features and GS cohorts were correlated using Spearman’s correlation.

The Kruskal-Wallis test showed that the three GS cohorts (G2, G3, and G4) were statistically different in uniformity (Table 4). After applying the Holm-Bonferroni correction, no other characteristics were significantly different between GS groups. The correlation coefficients for entropylog2, entropylog10, uniformity and the angular second moment are 0.23, 0.23, −0.24, and −0.26. These numbers have a low correlation (Table 5).

### 3.5. Prediction of Gleason Score

**Figure 4:**
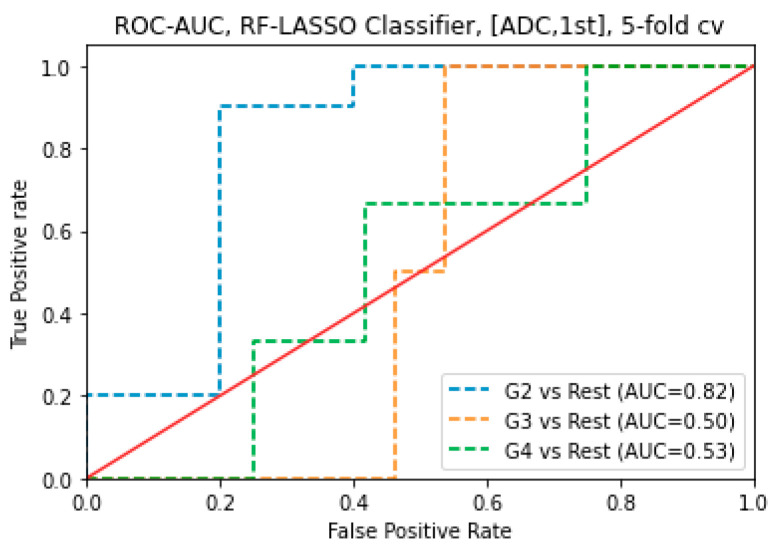
ROC-AUC of predicting GS of prostate cancer from RF classifier (using LASSO feature selections) using 1st order features obtained from ADC map images.

The RF and SVM classifiers with tuning hyperparameters model predicted the GS groups of 71 prostate cancer subjects using all radiomics features. For ADC map images, using 1st order features, the LASSO combined RF Classification algorithm was an AUC of 0.82 for G2 subjects, 0.53 for G4 subjects, and 0.50 for G3 subjects. The RFE combined RF Classification algorithm was an AUC of 0.77 for G3 subjects, 0.71 for G3 subjects, and 0.43 for G4 subjects. The RFE combined SVM Classification algorithm was an AUC of 0.81 for G3 subjects, 0.48 for G2 subjects, and 0.25 for G4 subjects. The LASSO combined SVM Classification algorithm was an AUC of 0.77 for G4 subjects, 0.40 for G2 subjects, and 0.22 for G4 subjects. For ADC map images, using 1st order features, the LASSO with RF classification algorithm obtained the highest AUC of 0.82 to predict G2 (Figure 4).

**Figure 5:**
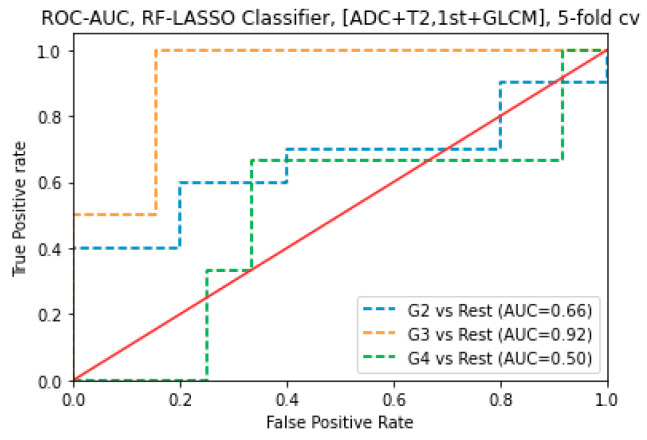
ROC-AUC of predicting GS of prostate cancer from SVM classifier (using LASSO feature selections) using GLCM features obtained from T_2_WI images.

For combined sequences (T_2_WI and ADC map images) and features (1st order and GLCM), the LASSO combined RF Classification algorithm had an AUC of 0.92 for G3 subjects. For combined sequences (T_2_WI and ADC map images) and features (1st order and GLCM), the RFE combined RF Classification algorithm was AUC of 0.73 for G3 subjects and 0.61 for G4 subjects, respectively and 0.54 for G2 subjects. For combined sequences (T_2_WI and ADC map images) and features (1st order and GLCM), the LASSO combined SVM classification algorithm was an AUC of 0.78 for G4 subjects, 0.65 for G3, and 0.62 for G2 subjects. For combined sequences (T_2_WI and ADC map images) and features (1st order and GLCM), the RFE combined SVM. For combined sequences (T_2_WI and ADC map images), the LASSO with RF classification algorithm obtained the maximum AUC of 0.92 to predict G3 (Figure 5). 

**Figure 6:**
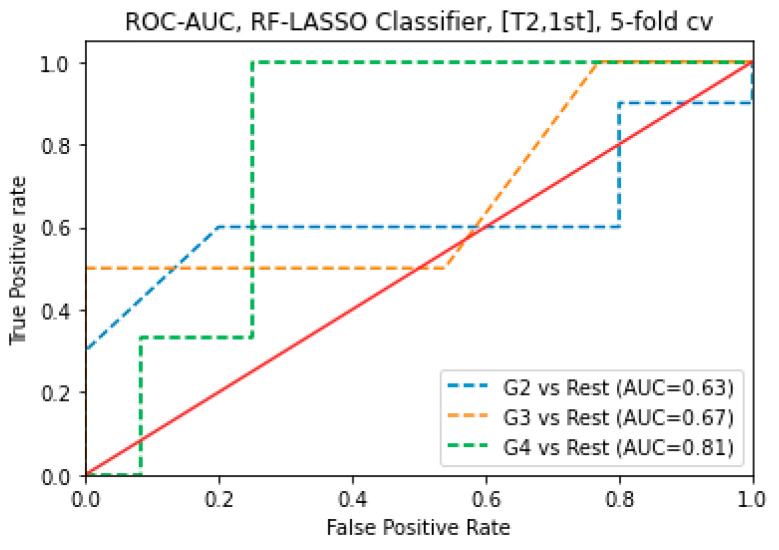
ROC-AUC of predicting GS of prostate cancer from RF classifier (using LASSO feature selections) using 1st order features obtained from T_2_WI image.

For T_2_WI images, using 1st-order features, the LASSO combined RF Classification algorithm was an AUC of 0.81 for G4 subjects, 0.67 for G3 subjects, and 0.63 for G2 subjects. For T_2_WI images, using 1st order features, the LASSO with RF classification algorithm obtained the highest AUC of 0.81 to predict G4 (Figure 6).

## 4. Discussion

In PCa assessment, mp-MRI has been demonstrated to be a superior technique, allowing for greater accuracy when detecting cancerous growths. That is the only imaging approach with enough spatial resolution and soft tissue contrast to identify prostate cancer effectively [8] without using ionising radiation. Prostate tumor aggressiveness can be evaluated using artificial intelligence, such as radiomics [57]. Consequently, radiomics could be an innovative and effective method for extracting further clinically relevant data [17]. Radiomics can diagnose prostate cancer early, grade it according to Gleason, determine therapy response, and anticipate biochemical recurrence [57]. 

Different clinical settings may require different ML techniques for discriminating between sacral chordoma and sacral giant cell malignancies; LASSO using a generalised linear model (GLM) significantly outperformed [29,48]. However, when it came to scoring colon microarray gene expression and identifying meningioma, random forest and eXtreme Gradient Boosting (XGBoost) classification methods achieved the best performance [30,31,32,48,58]. Wang et al. revealed that the ML approach of recursive elimination features using a support vector machine is better than other feature selection and classification methods [48]. As a result, it is essential and recommended to discover appropriate machine learning approaches in various clinical implementations in future studies. In the context of prostate cancer classification and grading, our research stands out due to its focus on hyperparameter tuning. While many prior studies have applied machine learning techniques with default hyperparameters, we have systematically optimized these parameters to enhance the precision and robustness of our models. This approach has demonstrated its potential to contribute to more accurate and clinically relevant diagnoses, highlighting the critical role of hyperparameter optimization in medical applications of machine learning.

### 4.1. Significant Cancer versus Non-Tumor Regions

The Kruskal-Wallis test was utilised to examine radiomics characteristics’ relevance in differentiating significant cancer versus non-tumor regions. Then, Spearman correlation was performed to determine the association between radiomics attributes and significant cancer versus non-tumor regions. Two feature selection methods (REF and LASSO) and two classifiers (RF and SVM) with tuned hyperparameters (randomised search and grid search) were used to create an effective ML algorithm. The analysis between radiomics features and the significant versus non-tumor regions revealed eleven radiomics features that are statistically significant (i.e., skewness, kurtosis, entropylog1o, entropylog2, uniformity, jointEntropyLog2, jointEntropyLog10, correlation, contrast, dissimilarity, and angular second moment) with the capacity to discriminate between the significant and non-tumor regions. Skewness and kurtosis reflect the distribution and shape of pixel intensities, indicating tissue composition or structural variations. Both entropylog1o and entropylog2 measure randomness in pixel distribution, revealing spatial tumor cell distribution. Uniformity indicates pixel intensity homogeneity, suggesting uniform tissue composition or density. Joint entropy reflects spatial relationship randomness, correlating with tumor heterogeneity. Correlation measures linear intensity relationships, indicating tissue structure homogeneity. Contrast reveals local intensity variations, suggesting distinct tumor features. Dissimilarity measures intensity differences between neighboring voxels, reflecting tissue heterogeneity. Angular second moment quantifies intensity uniformity, indicating tissue texture homogeneity. Overall, these features provide insights into tumor heterogeneity by quantifying pixel intensity distribution, texture, and spatial relationships within the tumor region.

Prostate cancer discrimination employing multiparametric MRI radiomics was designed and tested in this study, and the technique consistently performed well in the present study. As this study reveals, classification accuracy varies between ML techniques. For T_2_WI, RF and SVM classifiers were observed to be very useful when used with REF (AUC = 0.95 ± 0.01, and 0.94 ± 0.01, respectively). The second-best result was observed using LASSO selection with SVM and RF classifiers (AUC = 0.93 ± 0.01 for T_2_WI, and 0.89 ± 0.00 for ADC map, respectively). That is following previous findings have shown that this system is adequate to other feature selection techniques and classifiers in various organs [32,45,55,57,59,60]. With support vector machines and random forests classifiers, the AUC for the T_2_WI sequence was maximum with the selection approach using the REF. Radiomic features can be used to identify the T1-2 and T3-4 stages using an unsupervised clustering algorithm and the supervised LASSO technique, according to Sun et al. [61]. This finding might link to the fact that morphological T_2_WI depends on the tumor signal for its assessment. The second-highest AUC was achieved using the selection approach of LASSO with SVM and RF. Wang et al. achieved the best result when combining a support vector machine with recursive feature reduction [62]. 

Nevertheless, the T_2_WI model performed better than the ADC model (AUCs of 0.95 vs. 0.89, respectively). We observed that the AUC of the classification algorithm generated from T_2_WI images using RF classifiers using the feature selection technique (RFE) was the maximum AUC of 0.95 ± 0.01. In addition, the RFE combined with the SVM classification algorithm obtained the second maximum AUC of 0.94 ± 0.01 (with 5-fold cross-validation). Additionally, T_2_WI could perform a non-invasive analysis of PCa biological growth, which might assist in classifying patients for adequate treatment. It also provides morphologic data for cancer diagnosis, localisation, and staging [62]. SVM and RF classifiers combined LASSO (For LASSO, AUC of 0.89 ± 0.00, 0.89 ± 0.02 for SVM, and RF classifiers, respectively) and RFE (for RFE, AUC of 0.84 ± 0.01, 0.85 ± 0.02 for SVM, and RF classifiers, respectively) for classification between significant cancer versus non-tumor from ADC map images were lower when compared to T_2_WI images. For combined sequences (T_2_WI and ADC map images), the LASSO combined SVM classification algorithms had an AUC of 0.91. The second-highest AUC was 0.88 for the RFE with the RF classification algorithm. Features from several sequences achieved lower performance compared to single sequence features. 

### 4.2. GS Prediction

The Kruskal-Wallis test assessed radiomics features’ ability to predict GS in prostate cancer patients. Radiomics attributes and GS cohorts were then correlated using Spearman correlation. The ML algorithm was developed using feature selection methods (REF and LASSO) and classifiers (RF and SVM) with tuned hyperparameters (randomised search and grid search). ADC map images revealed one radiomics feature from the uniformity that could distinguish GS cohorts. Uniformity refers to the homogeneity of pixel intensities, indicating a consistent tissue composition or density. This feature offers insights into tumor heterogeneity by assessing the distribution of pixel intensities, texture variations, and spatial relationships within the tumor region, providing a comprehensive view of its internal characteristics.

For combined sequences (T_2_WI and ADC map images), the LASSO combined RF classification algorithm obtained the maximum AUC of 0.92 to predict G3 related to G4 (SVM-LASSO, AUC = 0.78) and G2 (RF-LASSO, AUC = 0.66). In addition, for T_2_WI images, using GLCM features, the LASSO combined SVM classification algorithm obtained the highest AUC of 0.92 to predict G3 related to G2 (RF-LASSO, AUC = 0.56) and G4 (SVM-LASSO, AUC = 0.61). Furthermore, for ADC map images, using the 1st order features, the LASSO with RF classification algorithm obtained the maximum AUC of 0.82 to predict G2 related to G3 (SVM-RFE, AUC = 0.81) and G4 (SVM-LASSO, AUC = 0.53). Additionally, for T_2_WI images, using First-order features, the LASSO combined RF classification algorithm obtained the maximum AUC of 0.81 to predict G4 related to G2 (SVM-LASSO and RF-RFE, AUC = 0.66, and 0.66 respectively) and G4 (RF-RFE, AUC = 0.78). 

The results we obtained agree with those of several other studies using texture analysis [63,64]. Texture features, such as those of the first and second order derived from ADC and T_2_WI, and sample augmentation, were demonstrated to effectively achieve reasonably accurate classification of Gleason patterns [55]. Our findings align with employing the Gleason score as the primary criterion for differentiating benign from significant prostate tumors.

There are limitations identified in this research. There is a relatively low N number of patients. A significant subject cohort (raw dataset) is required to fully validate and optimize the performance for application in a clinical setting. We agree that there are limitations to this work and that in clinical settings there are compromises made on mismatched resolutions. Ideally all our data and all clinical data would be at the same resolution field strength etc. providing uniformity in data acquisition and this step could be avoided. Due to the nature of clinical MRI time and the time requirement of different sequences employed this mismatch of resolutions will persist for the near future.

## 5. Conclusions

Within the scope of this study, the classification of prostate cancer and prediction of GS groups using multiparametric MRI-based radiomics has been achieved. By prioritizing hyperparameter tuning, we have significantly improved the precision and dependability of our ML approaches. This work underscores the importance of meticulous parameter optimization in enhancing the accuracy of medical diagnoses. Radiomics analysis based on multiparametric MRI showed excellent results in discriminating non-tumor regions from significant prostate cancer results obtained. The results of the radiomics analysis, which depended on the multiparametric MRI, demonstrated superior outcomes in predicting between GS groups. Our approach suggests that using multiple features and classifiers with tuning hyperparameters provided a more clinically dependable method of identifying clinically relevant features. 

## Figures and Tables

**Table 1:** Multiparametric MRI sequence parameters.

Sequence Parameter	T_2_WI	ADC
Repetition time (ms)	5560	2700
Echo time (ms)	104	63
Flip angle (degrees)	160	90
Bandwidth (Hz/px)	200	1500
Phase FoV%	100	65.625
Slice thickness (mm)	3	3
Slice gap (mm)	3	3
Average	4	8
Phase encoding direction	Row	Row
Number of acquisitions	1	1

**Table 2:** Collating of radiomics parameters of significant cancer versus non-tumor regions.

Feature	Median (Interquartile 25th, 50th, and 75th Percentiles)		*p*
	Significant Cancer	Nontumor Regions	
ADC			
1st order			
Skewness	0.37 (0.02, 0.37, 0.69)	0.03 (−0.29, 0.03, 0.47)	0.001
Kurtosis	−0.49 (−0.85, −0.49, 0.29)	−0.54 (−0.86, −0.54, −0.12)	0.52
Entropylog1o	1.14 (1.09, 1.14, 1.19)	1.09 (1.05, 1.09, 1.14)	˂0.001
Entropylog2	3.80 (3.62, 3.80, 3.97)	3.62 (3.50, 3.62, 3.80)	˂0.001
Uniformity	0.07 (0.06, 0.07, 0.08)	0.08 (0.07, 0.08, 0.10)	˂0.001
GLCM			
JointEntropyLog2	6.18 (5.85, 6.18, 6.45)	6.05(5.83, 6.05, 6.21)	0.03
JointEntropyLog10	1.86 (1.79, 1.86, 1.94)	1.82(1.75, 1.82, 1.87)	0.006
Angular Second Moment	0.01 (0.01, 0.1, 0.01)	0.016 (0.014, 0.016, 0.019)	0.006
Contrast	145.64 (107.88, 145.64, 201.96)	84.02 (59.85, 84.02, 122.08)	˂0.001
Dissimilarity	9.30 (8.33, 930, 11.36)	7.51 (6.19, 7.51, 8.61)	˂0.001
Correlation	0.18 (0.06, 0.18, 0.35)	0.23 (0.09, 0.23, 0.39)	0.37
T_2_WI			
1st order			
Skewness	0.07 (−0.20, 0.07, 0.32)	0.15 (−0.20, 0.15, 0.43)	0.50
Kurtosis	−0.18 (−0.55, −0.018, 0.43)	−0.34 (−0.59, −0.34, 0.11)	0.18
Entropylog1o	1.30 (1.23, 1.30, 1.41)	1.06 (0.97, 1.06, 1.16)	˂0.001
Entropylog2	4.34 (4.11, 4.34, 4.69)	3.52 (3.24, 3.52, 3.85)	˂0.001
Uniformity	0.05 (0.04, 0.05, 0.06)	0.09 (0.07, 0.09, 0.12)	˂0.001
GLCM			
JointEntropyLog2	7.50 (6.89, 7.50, 8.16)	6.45 (5.95, 6.45, 7.12)	˂0.001
JointEntropyLog10	2.31 (2.12, 2.31, 2.50)	1.96 (1.79, 1.96, 2.14)	˂0.001
Angular Second Moment	0.006 (0.004, 0.006, 0.01)	0.01 (0.01, 0.01, 0.02)	˂0.001
Contrast	92.42 (64.22, 92.42, 132.48)	13.36 (10.08, 13.36, 20.47)	˂0.001
Dissimilarity	7.62 (6.36, 7.62, 9.07)	2.88 (2.46, 2.88, 3.60)	˂0.001
Correlation	0.25 (0.13, 0.25, 0.35)	0.38 (0.24, 0.38, 0.50)	˂0.001

**Table 3:** Features related with the significant malignancy and the non-tumor regions are considered correlated.

Feature	r	*p*
ADC		
1st order		
Skewness	0.315	˂0.001
Entropylog1o	0.305	˂0.001
Entropylog2	0.305	˂0.001
Uniformity	−0.331	˂0.001
GLCM		
Angular Second Moment	−0.236	0.005
Contrast	0.376	˂0.001
Dissimilarity	0.468	˂0.001
T2WI		
1st order		
Entropylog1o	0.561	˂0.001
Entropylog2	0.561	˂0.001
Uniformity	−0.254	0.002
GLCM		
JointEntropyLog2	0.270	0.001
JointEntropyLog10	0.269	0.001
Contrast	0.765	˂0.001
Dissimilarity	0.809	˂0.001
Correlation	0.370	˂0.001

**Table 4:** Collating of radiomics parameters of PCa that are related with the GS.

Feature	Gleason Score Median (Interquartile 25th, 50th, and 75th Percentiles)			*p*
	G2	G3	G4	
ADC				
1st order				
Skewness	0.30 (−0.01, 0.30, 0.58)	0.60 (−0.12, 0.60, 1.24)	0.39 (0.10, 0.39, 0.75)	0.92
Kurtosis	−0.49 (−0.87, −0.49, 0.26)	−0.38 (−0.78, −0.38, 1.24)	−0.34 (−0.90, −0.34, 0.49)	0.81
Entropylog1o	1.12 (1.08, 1.12, 1.16)	1.15 (1.09, 1.15, 1.21)	1.16 (1.09, 1.16, 1.22)	0.03
Entropylog2	3.75 (3.61, 3.75, 3.87)	3.83 (3.62, 3.83, 4.03)	3.88 (3.64, 3.88, 3.06)	0.03
Uniformity	0.07 (0.07, 0.07, 0.08)	0.07 (0.06, 0.07, 0.08)	0.07 (0.06, 0.07, 0.08)	0.01
GLCM				
JointEntropyLog2	6.12 (5.87, 6.12, 6.47)	7.84 (7.42, 7.84, 8.22)	6.28 (6.11, 6.28, 6.60)	0.03
JointEntropyLog10	1.84 (1.76, 1.84, 1.94)	2.36 (2.24, 2.36, 2.54)	1.89 (1.83, 1.89, 1.98)	0.18
Angular Second Moment	0.02 (0.01, 0.02, 0.02)	0.005 (0.0037, 0.005, 0.006)	0.01 (0.01, 0.01, 0.01)	0.05
Contrast	132.43 (101.12, 132.43, 182.76)	83.79 (64.25, 83.79, 128.98)	149.88 (107.82, 149.88, 220.89)	0.15
Dissimilarity	9.01 (8.05, 9.01, 10.79)	7.09 (6.25, 7.09, 9.03)	9.84 (8.25, 9.84, 11.96)	0.14
Correlation	0.18 (0.02, 0.18, 0.33)	0.26 (0.14, 0.26, 0.47)	0.20 (0.05, 0.20, 0.41)	0.54
T_2_WI				
1st order				
Skewness	0.03 (−0.22, 0.03, 0.29)	0.23 (−0.12, 0.23, 0.47)	−0.03 (−0.26, −0.03, 0.23)	0.85
Kurtosis	−0.14 (−0.47, −0.14, 0.64)	0.07 (−0.39, 0.07, 0.27)	−0.62 (−0.76, −0.62, −0.31)	0.78
Entropylog1o	1.29 (1.23, 1.29, 1.38)	1.33 (1.26, 1.33, 1.44)	1.28 (1.18, 1.28, 1.38)	0.76
Entropylog2	4.31 (4.09, 4.31, 4.61)	4.42 (4.20, 4.42, 4.47)	4.28 (3.92, 4.28, 4.61)	0.76
Uniformity	0.05 (0.04, 0.05, 0.06)	0.05 (0.04, 0.05, 0.06)	0.05 (0.04, 0.05, 0.07)	0.80
GLCM				
JointEntropyLog2	7.48 (6.87, 7.48, 8.16)	6.17 (5.75, 6.17, 6.41)	7.12 (6.79, 7.12, 8.23)	0.40
JointEntropyLog10	2.28 (2.11, 2.28, 2.25)	1.88 (1.76, 1.88, 2.01)	2.19 (2.06, 2.19, 2.48)	0.72
Angular Second Moment	0.008 (0.004, 0.01, 0.01)	0.01 (0.01, 0.01, 0.02)	0.01(0.01, 001, 0.01)	0.69
Contrast	94.93 (69.61, 94.93, 138.60)	180.96 (126.01, 180.96, 280.64)	101.25 (54.24, 101.25, 125.81)	0.62
Dissimilarity	7.68 (6.58, 7.68, 9.03)	9.71 (8.62, 9.71, 13.23)	7.97 (6.06, 7.97, 9.17)	0.63
Correlation	0.24 (0.10, 0.24, 0.34)	0.24 (0.07, 0.24, 0.33)	0.22 (0.14, 0.22, 0.31)	0.78

**Table 5:** Features that relate to the GS are considered correlates.

Feature	r	*p*
ADC		
1st order		
Uniformity	−0.30	0.02

## Data Availability

The data presented in this study is available upon request from the corresponding author, while the data is open access.
